# Novel biphenylidene-thiopyrimidine derivatives as corrosion inhibitors for carbon-steel in oilfield produced water

**DOI:** 10.1038/s41598-023-43312-6

**Published:** 2023-09-29

**Authors:** Hajar A. Ali, Mahmoud M. Shaban, Ashraf S. Abousalem, Eslam A. Ghaith, Abdelaziz S. Fouda, Mohamed A. Ismail

**Affiliations:** 1 Chemistry Department, Faculty of Science, Mansoura University, Mansoura, 35516 Egypt; 2 Egyptian Petroleum Research Institute, Nasr City 11727, Cairo, Egypt; 3 Quality Control Laboratory, Operations Department, Jotun, Egypt

**Keywords:** Chemical physics, Materials science

## Abstract

The inhibiting efficiency of three newly synthesized organic compounds:5-((4'-(dimethylamino)-[1,1'-biphenyl]-4-yl)methylene)-1,3-diethyl-2-thioxodihydropyrimidine-4,6(1*H*,5*H*)-dione (HM-1228), 5-((4'-(dimethylamino)-[1,1'-biphenyl]-4-yl)methylene)-2-thioxodihydropyrimidine-4,6(1*H*,5*H*)-dione (HM-1227) and 5-((4'-(dimethylamino)-[1,1'-biphenyl]-4-yl)methylene)pyrimidine-2,4,6(1*H*,3*H*,5*H*)-trione (HM-1226) in oilfield produced water on the corrosion of carbon steel has been examined via electrochemical measurements; potentiodynamic polarization (PDP) and electrochemical impedance (EIS) techniques. The adsorption of these compounds on the surface of carbon steel followed Langmuir isotherm. In addition, the surface morphology of uninhibited and inhibited carbon steel was examined by Atomic Force Microscopy (AFM), observing surface improvement when carbon steel samples exposed to the inhibited corrosive solutions. The average surface roughness (*Ra*) in oilfield produced water solution in the presence of 0.5 mM of HM-1228 inhibitor was 138.28 nm compared to the uninhibited surface 571.62 nm. To explore the corrosion inhibition mechanism, quantum chemical calculations and Monte Carlo simulations were utilized. The HM-1228 inhibitor demonstrated the highest corrosion inhibition efficiency at 94.8% by PDP measurements. The higher corrosion inhibition of compound HM-1228 can be attributed to the presence of di-*N*-ethyl groups that enhance both electron donating ability and lipophilic properties.

## Introduction

Carbon steel is one of the most widely used construction materials in various industries, such as oil and gas transportation, chemical, and petrochemical. Its alloys are particularly prevalent due to their unique mechanical and machinery properties, including hardness, strength, and ductility. However, a major challenge associated with the use of carbon steel alloys is their lack of corrosion resistance, especially in moist conditions. This can be considered as a drawback as it can lead to significant industrial problems and jeopardize the safety of humans. Therefore, there has been a growing interest among researchers and industry specialists in finding solutions to mitigate corrosion, focusing on corrosion protection measures to control these undesired consequences and prolong the lifespan of structural materials^1,2^. Since the useful features of carbon steel can compromised by severe attack from the corrosive environment of various oilfield operations^3–5^. One of the most prevalent corrosive environments in the crude oil and gas industry is oil-well formation water, which occurs naturally in oil fields and contain a variety of dissolved inorganic and organic chemicals, dissolved salts, and dissolved gases^6,7^. One viable approach for industry experts to mitigate corrosion is the use of corrosion inhibitors which protect the steel surface via the formation of a protective film. The use of corrosion inhibitors, which is a highly effective industry technique, is commonly utilized to reduce metal loss^8–10^. Some organic compounds are synthesized and used as corrosion inhibitors due to their several features including the low price, high inhibition efficacy, and ease of synthesis^11–14^. However, the use of organic compounds as corrosion inhibitors is one of the most challenging tasks because most organic compounds are neither cheap nor effective in most cases. It is generally established that organic compounds including sulphur, oxygen, and nitrogen atoms protect the steel surface from the destructive media via adsorption^15^. In a previous work in literature, the thiobarbituric acid was tested for its inhibition efficiency for API X60 steel in NaCl solution saturated with CO_2_, which showed corrosion inhibition above 90%^16^. In recent years, ongoing efforts have been devoted to synthesize new, more efficient corrosion inhibitors, which is an important direction in metal corrosion prevention.

Even though previous studies on thiopyrimidine derivatives have been published as corrosion inhibitors, our aim in this study is to investigate the inhibition efficiency of novel biphenylidene-thiopyrimidine derivatives as corrosion inhibitors for carbon steel in oilfield produced water using a combination of experimental and computational chemistry approaches.

## Experimental

Three novel biphenylidene-thiopyrimidine derivatives HM-1228, HM-1227, and HM-1226 were synthesized. The chemical structures and molecular formulas are presented in Table 1. The full details on the synthesis process and characterization are in the following experimental part.

**Table 1:** Molecular structures, formulas and weights of the investigated inhibitors.

Code	Structures/Names	Formulas (F. wt.)
HM-1228	5-((4'-(Dimethylamino)-[1,1'-biphenyl]-4-yl)methylene)-1,3-diethyl-2-thioxodihydropyrimidine-4,6(1*H*,5*H*)-dione	C_23_H_25_N_3_O_2_S (407.53)
HM-1227	5-((4'-(Dimethylamino)-[1,1'-biphenyl]-4-yl)methylene)-2-thioxodihydropyrimidine-4,6(1*H*,5*H*)-dione	C_19_H_17_N_3_O_2_S (351.42)
HM-1226	5-((4'-(Dimethylamino)-[1,1'-biphenyl]-4-yl)methylene)pyrimidine-2,4,6(1*H*,3*H*,5*H*)-trione	C_19_H_17_N_3_O_3_ (335.36)

### Methodology for preparation of the studied inhibitors

#### Preparation of (*N*,*N*-dimethylamino)biphenylidene-thiopyrimidine derivatives 5a-c:

4^\^-(*N*,*N*-Dimethylamino)-[1,1^\^-biphenyl]-4-carbaldehyde (**3**): To a solution of 4-bromo-*N*,*N*-dimethylaniline (**1**) (1.5 g, 7.5 mmol), and Pd(PPh_3_)_4_ (230 mg) in toluene (15 mL) was added 7.5 mL of 2M Na_2_CO_3_ (aqueous), and methanolic solution of (4-formylphenyl)boronic acid **2** (1.35 g, 9 mmol). The reaction mixture was heated at 80 °C with stirring for ~ 12 h, where after the reaction mixture was extracted with ethyl acetate (200 mL, 3x). The organic layer was passed through celite to remove Pd, the organic extract was dried and then evaporated to dryness. The solid was filtered off and recrystallized from ethanol to furnish the anticipated 4^\^-(dimethylamino)-[1,1^**\**^-biphenyl]-4-carbaldehyde **3**. Biphenylcarbaldehyde **3** was obtained in 82% yield as a yellow solid, m.p. = 189–191 °C, Lit.^17^ m.p. = 192–195 °C. IR (KBr) ν^\^/cm^−1^: 3035 (sp^2^ C–H, stretch), 2892 (sp^3^ C–H, stretch), 2805, 2713 (C–H of CHO, stretch), 1693 (C = O, stretch), 1594, 1541 (C = C, stretch). MS (EI) m/e (rel.int.) for C_15_H_15_NO (225.29); 225.39 (M^+^, 42.24), 77 (100).

#### 5-((4'-(Dimethylamino)-[1,1'-biphenyl]-4-yl)methylene)-1,3-diethyl-2-thioxodihydro-pyrimidine-4,6(1*H*,5*H*)-dione (5a)

To a solution of biphenylcarbaldehyde **3** (200 mg, 0.89 mmol), 1,3-diethyl-2-thiobarbituric acid (**4a**) (1.78 mmol) in a mixture of 30 mL methanol/acetic acid (2:1). The reaction mixture was heated at reflux for ~ 12 h and the precipitate was filtered off, washed with methanol and recrystallized from ethanol/Ethyl acetate to afford biphenylidene-thiobarbituric acid derivative **5a** as a greenish-brown solid in 71% yield, m.p. = 216–218 °C. IR (KBr) ν^**\**^/cm^−1^: 2974, 2927 (sp^3^ C–H, stretch), 1697, 1664 (C = O, stretch), 1598, 1554 (C = C, stretch), 1396 (C = S, stretch). ^1^H-NMR (DMSO-*d*_6_); *δ* 1.19–1.22 (m, 6H, 2CH_3_ of diethyl groups-H’s), 2.97 (s, 6H, –N(CH_3_)_2_), 4.40–4.44 (m, 4H, 2CH_2_ of diethyl groups-H’s), 6.81 (d, *J* = 9 *Hz*, 2H, Ar–H’s of dimethylaniline ring), 7.71 (d, *J* = 9 *Hz*, 2H, Ar–H’s of phenyl ring), 7.78 (d, *J* = 9 *Hz*, 2H, Ar–H’s of phenyl ring), 8.29 (d, *J* = 9 *Hz*, 2H, Ar–H’s of dimethylaniline ring), 8.38 ppm (s, 1H, methine-H). MS (EI) m/e (rel.int.) for C_23_H_25_N_3_O_2_S (407.53); 407.41 (M^+^, 25.89), 150.56 (100).

#### 5-((4'-(Dimethylamino)-[1,1'-biphenyl]-4-yl)methylene)-2-thioxodihydropyrimidine-4,6(1*H*,5*H*)-dione (5b)

Compound **5b** was prepared adopting the same methodology used for preparation of compound **5a**, starting with 2-thiobarbituric acid (**4b**) instead of 1,3-diethyl-2-thiobarbituric acid (**4a**). Thiopyrimidine derivative **5b** was obtained as a greenish-brown solid in 75% yield, m.p. > 300 °C. IR (KBr) ν^**\**^/cm^−1^: 3451, 3419 (N–H, stretch), 3158 (sp^2^ C–H, stretch), 2926 (sp^3^ C–H, stretch), 1697, 1652 (C = O), 1612, 1523 (C = C, stretch), 1395 (C = S, stretch). ^1^H-NMR (DMSO-*d*_6_); *δ* 2.97 (s, 6H, –N(CH_3_)_2_), 6.80 (d, *J* = 8.5 *Hz*, 2H, Ar–H’s of dimethylaniline ring), 7.71 (d, *J* = 8.0 *Hz*, 2H, Ar–H’s of phenyl ring), 7.77 (d, *J* = 8 *Hz*, 2H, Ar–H’s of phenyl ring), 8.27 (s, 1H, methine-H), 8.32 (d, *J* = 8.5 *Hz*, 2H, Ar–H’s of dimethylaniline ring), 12.33 (s, 1H, NH), 12.43 ppm (s, 1H, NH). ^13^CNMR; *δ* 39.5 (2C), 112.6 (2C), 117.1, 124.6, 127.7 (2C), 129.9 (2C), 130.2 (2C), 135.6, 145.0, 150.6, 155.6, 159.8, 162.1, 178.3. MS (EI) (m/e, %) for C_19_H_17_N_3_O_2_S (351.42); 351.53 (M^+^, 28.96), 93.34 (Base peak, 100).

#### 5-((4'-(Dimethylamino)-[1,1'-biphenyl]-4-yl)methylene)pyrimidine-2,4,6(1*H*,3*H*,5*H*)-trione (5c)

Compound **5c** was furnished adopting the same methodology used for preparation of **5a**, starting with barbituric acid (**4c**) instead of 1,3-diethyl-2-thiobarbituric acid (**4a**). Compound **5c** was obtained as a greenish-brown solid in 72% yield, m.p. = 250–252 °C. IR (KBr) ν^**\**^/cm^−1^: 3201 (N–H, stretch), 3060 (sp^2^ C–H, stretch), 2851 (sp^3^ C–H, stretch), 1753, 1701, 1658 (C = O, stretch), 1596, 1547 (C = C, stretch). ^1^H-NMR; *δ* 2.96 (s, 6H, –N(CH_3_)_2_), 6.80 (d, *J* = 8.5 *Hz*, 2H, Ar–H’s of dimethylaniline ring), 7.69 (d, *J* = 8.5 *Hz*, 2H, Ar–H’s of phenyl ring), 7.75 (d, *J* = 8.5 *Hz*, 2H, Ar–H’s of phenyl ring), 8.26–8.28 (m, 3H), 11.22 (s, 1H, NH), 11.35 ppm (s, 1H, NH). MS (EI) m/e (rel.int.) for C_19_H_17_N_3_O_3_ (335.36); 335.94 (M^+^, 38.79), 334.95 (M^+^ − 1, 100).

### Corrosion measurements

Corrosion experiments were performed using API 5L X52 carbon steel specimens with weight percentage element composition shown as follows: C (0.114), Si (0.346), Mn (0.967), Ni (0.071), Cr (0.163), Al (0.043), Cu (0.124), Nb (0.053), Pb (0.034), Ti (0.04) and Fe (98.027). The aggressive media utilized in this study is oilfield produced water obtained from oil well of Badr El-Din Petroleum Company, Egypt. Physical properties and chemical composition of the oil field produced water are presented in Table 2. The equivalent weight of inhibitor was dissolved in 10 ml dimethyl-sulfoxide then the volume was completed with absolute ethanol up to 100 ml. The concentration ranges of the studied inhibitors were 0.001–0.5 mM. Electrochemical studies (PDP and EIS) were performed by a three-electrode electrochemical glass cell using Volta lab 80. API 5L X52 carbon steel with an uncovered surface area of 0.8 cm^2^ was utilized as the working electrode, Pt plate was served as the counter electrode, and Ag/AgCl/KCl_sat_ electrode was employed as reference electrode. Prior to measurements, the working electrode was polished to mirror in series on 360–2500 silicon carbide papers, then washed with deionized water and dimethyl ketone, and finally dried under warm air. EIS experiments were carried out in a frequency range between 10^5^ Hz and 10^─2^ Hz at open circuit potential (*E*_OCP_), with a signal (AC) amount perturbation of 10 mV^18,19^. PDP tests were evaluated at ± 300 mV based on *E*_OCP_ with a steady sweep rate of 1 m mV s^−1^^20,21^. Each electrochemical test had been executed at least twice to confirm the accuracy of the data.

**Table 2:** Physical properties and chemical composition of the deep oil wells formation water containing sulfide ions used in this work.

Physical properties		
Property	Unit	Value
Density	g/cm^3^	1.044
Turbidity	FAU	263
pH		7.66
Specific gravity		1.0050
Salinity as NaCl	ppm (mg/l)	24075
Iron	ppm	3.76
Total alkalinity	ppm	260
Total hardness	ppm	604
Temporary hardness	ppm	260
Permanent hardness	ppm	344
Conductivity	μS/cm	26220
Chemical properties		
Ionic species	Unit	Value
Sodium & Potassium	ppm	1577
Calcium	ppm	168
Magnesium	ppm	45
Barium	ppm	0.13
Strontium	ppm	8.22
Chlorides	ppm	24720
Sulphate	ppm	577
Bicarbonates	ppm	317
Carbonates	ppm	35
T.D.S	ppm	7000
Barium Sulphate	ppm	0.2
Calcium Carbonate	ppm	260
Strontium	ppm	17
Calcium Sulphate	ppm	217
Magnesium Sulphate	ppm	233
Sod. & Pot. Chlorides	ppm	4448

#### Surface morphology investigations

Surface analysis (AFM) of API 5L X52 carbon steel specimens, with dimensions 15 × 10 × 2.5 mm, was performed by Nano surf Flex AFM analysis without and with exposure to oil field produced water solution without and with 0.5 mM of the prepared inhibitor HM-1228 at 25 ± 1 °C. Carbon steel samples were stored, and then dried for AFM analysis^10,22^.

#### Computational calculations

The ground-state geometry optimizations were carried out with Density Function (DFT) method on the investigated biphenylidene-thiopyrimidine derivatives by Gaussian 09, Revision A02^23,24^. Prior to the geometrical optimization step, the most stable configuration of compounds is chosen and relaxed with MM2 force field tool. The DFT calculations were computed at basis-set 6-31G (d,p) and in absence of symmetry limitations and the Becke three parameter-hybrid-B3^25^ with the L-Y-P (Lee–Yang–Parr) correction functional^26^ (B3LYP)^27^. For the representation of solvent effect, the Polarized continuum (PCM) model was adopted. The quantum chemical parameters such as E_HOMO_, E_LUMO_, ΔE were calculated by DFT and analyzed^28^. The theoretical formulation of the HSAB principle is an effective approach to obtain some quantum chemical descriptors^29,30^. This method is based on the first and second partial derivatives of energy with regards to electron number at constant external potential.

Insightful data on the nature of chemical interactions and chemical reactivity of molecules can be studied, where some useful quantum chemical descriptors such as chemical hardness (η), chemical potential (μ), and electronegativity (χ) are evaluated. μ and η are calculated from the first derivation of the electronic energy and chemical potential as a function of electron number (*N*) at constant external potential, *v*_(r)_, respectively^31,32^.

1 $$\eta = \frac{1}{2}\left( {\frac{{\partial^{2} E}}{{\partial N^{2} }}} \right)_{\nu \left( r \right)} = \frac{1}{2}\left( {\frac{\partial \mu }{{\partial N}}} \right)_{\nu \left( r \right)}$$

2 $$\mu = \left( {\frac{\partial E}{{\partial N}}} \right)_{\nu \left( r \right)}$$

The HOMO and LUMO orbital energies correlates with the ionization energies (Eq. 3) and electron affinity of a molecule (Eq. 4)according to Koopman's Theorem^33^.

3 $$I = - E_{{{\text{HOMO}}}}$$

4 $$A = - E_{{{\text{LUMO}}}}$$

The hardness $\eta$, electronegativity $\chi$ and chemical potential ($\mu$ are also calculated by applying the following equations.

5 $$\chi \left( {Electronegativity} \right) = - \mu \left( {Chemical\; potential} \right) = \left( {\frac{{{\text{I}} + {\text{A}}}}{2}} \right)$$

6 $$\eta\,\left( {Hardness} \right) = \left( {\frac{{E_{{{\text{LUMO}}}} - E_{{{\text{HOMO}}}} }}{2}} \right)$$

Softness is the inverse of chemical hardness, and it can be used to indicate the polarizability of the molecule and it is given as the following equation:

7 $$\sigma \left( S \right) = 1/\eta$$

The global electrophilicity index (by Parr) (ω)^34^ quantifies the propensity of chemical species to accept electrons. A nucleophile with higher reactivity is distinguished by lower values of µ and ω, whereas an electrophile with higher reactivity is characterized by a higher value of µ and ω. This novel reactivity index evaluates the energy stabilization that occurs when the system obtains an extra electronic charge ∆N from its surroundings.

The global electrophilicity index (ω), nucleophilicity (ε), the fraction of electrons transferred (Δ*N*_*FET*_), the back donation energy Δ*E*_b-d_, electronic charge accepting capability and the initial molecule–metal interaction energy Δψ, were calculated in terms of global hardness (η) and electronegativity (χ) are calculated as the following Eqs. ^34^.

8 $$\omega \left( {Electrophilicity} \right) = \mu^{2} /2\eta = \chi^{2} /2\eta$$

9 $$\varepsilon \left( {Nucleophilicity} \right) = 1/\omega$$

10 $$\Delta E_{{\text{back - donation}}} = - \frac{\eta }{4} = \frac{1}{8}\left( {E_{{}} - E_{{{\text{LUMO}}}} } \right)$$

11 $${\Delta }N_{{{\text{FET}}}} = \frac{{\varphi_{{{\text{Fe}}}} - \chi_{inh} }}{{2\left( {\eta_{{{\text{Fe}}}} + \eta_{{{\text{inh}}}} } \right)}}$$

Herein, ${\varphi }_{\mathrm{Fe}}$ represents the work function of a metal surface, which is recently used as a measure of its electronegativity, and the ${\eta }_{\mathrm{Fe}}$ is absolute hardness of iron. Whereas ${\chi }_{\mathrm{inh}}$, and ${\eta }_{\mathrm{inh}}$ denote to the absolute electronegativity and hardness of inhibitor molecule, respectively. In order to obtain the ΔN values, we use a theoretical value of ${\varphi }_{\mathrm{Fe}}$ = 4.82 eV and ηFe = 0 by assuming that for a metallic bulk I = A since they are softer than the neutral metal atoms^35^.

#### Molecular simulations

Molecular simulations by Monte Carlo method which is available in adsorption locator tool in Materials Studio 7.0 is utilized to explore the lowest configuration adsorption energy on simulated inhibited and uninhibited Fe-surface in aqueous solution^36–38^. A simulation box with dimension 24.18 × 24.18 × 42.65 cm was used in the simulation procedure. First, the Fe (110) was cleaved from pure Fe crystal, then the surface was enlarged to a super cell 8 × 8 to accommodate the inhibitors on Fe surface. A vacuum slap with thickness 30 cm is built over the surface and the top layer surface atoms are dimensionally constrained. COMPASS force field is used in the simulation of corrosion inhibition process, as it is a high quality forcefield that enables consolidating frameworks of inorganic and organic compounds^37^. The tolerance of energy convergence is 10^−4^ kcal/mol with maximum force 0.005 kcal/mol/Å, and displacement of 5 × 10^−5^ Å. All the adsorption simulations are carried out in fine quality conditions. The simulation box contains Fe -surface layer and 1 inhibitor molecule, and 100 water molecules. The results from simulation were analyzed and discussed.

## Results and discussion

### Synthesis and characterization of inhibitors

**Figure 1:**
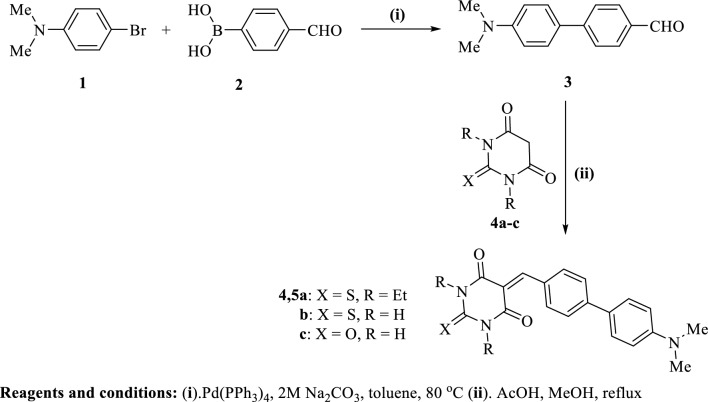
Synthesis for the new biphenylidene-thiobarbituric acid derivatives **5a–c**.

Preparation of the new biphenylidene-thiopyrimidine derivatives **5a-c** began with a Suzuki coupling by treatment of 4-bromo-*N,N*-dimethylaniline (**1**) with (4-formylphenyl)boronic acid **2** in the presence of Pd(0) as a catalyst and 2M Na_2_CO_3_ (aqueous) as a base and heating in toluene at 80 °C to furnish the biphenylcarbaldehyde compound **3** which is subsequently condensed with thiobarbituric acid derivatives **4a–c** to afford the target thiopyrimidine derivatives **5a–c** in good yields (71–75%) Fig. 1.

The newly synthesized biphenylidene-thiopyrimidines **5a–c** were assured based on their spectral data. Thus, IR spectrum of **5a** revealed no N–H absorption band, whereas, **5b** and **5c** indicated the appearance of N–H absorption bands at 3451, 3419 cm^−1^ (**5b**), 3201 cm^−1^ (**5c**), in addition to, thione groups at 1396 cm^−1^ (**5a**), 1395 cm^−1^ (**5b**). Whereas, IR spectra for compounds **5a–c** showed carbonyl groups in the range of 1652 to 1753 cm^−1^. ^1^H-NMR spectrum of compound 5a displayed the protons of diethyl groups as two multiplets at *δ* 1.19–1.22 (6H, 2 × CH_3_ of diethyl groups-H’s), 4.40–4.44 (4H, 2 × CH_2_ of diethyl groups-H’s), dimethylamino group (2 × N-Me) as a singlet signal at *δ* 2.97 integrated for six hydrogens, four aromatic hydrogens of dimethylaniline ring as two doublets at *δ* 6.81 (2H) and *δ* 8.29 (2H) with coupling constant *J* = 9 *Hz*, four aromatic hydrogens of phenyl ring as two doublets at *δ* 7.71 (2H) and *δ* 7.78 (2H), plus one singlet signal of methylidene at *δ* 8.38 (1H). Mass spectrum of **5a** furnished m/e peak at 407.41 of its molecular ion peak (M^+^, 25.89). ^1^H-NMR spectrum of compound **5b** displayed a singlet signal at *δ* 2.97 integrated for six hydrogens (2 × N-Me), four doublet signals; two of them referring to Ar–H’s of dimethylaniline ring at *δ* 6.80 (2H) and *δ* 8.32 (2H) with coupling constant *J* = 8.5 *Hz*, while other two doublets at *δ* 7.71 (2H) and *δ* 7.77 (2H) with coupling constant *J* = 8 *Hz* of Ar–H’s of phenyl ring, one singlet signal of methylidene at *δ* 8.27 (1H), plus two singlet signals integrated for one hydrogen each at *δ* 12.33 (NH) and 12.43 (NH). Mass spectrum of **5b** furnished peak at m/e 351.53 of its M^+^. Once more, ^1^H-NMR spectrum of compound **5c** displayed a singlet signal at *δ* 2.96 integrated for six hydrogens (2 × N-Me), one doublet signal at *δ* 6.80 integrated for two hydrogens (Ar–H’s of dimethylaniline ring), two doublet signals at *δ* 7.69 (2H) and *δ* 7.75 (2H) referring to Ar–H’s of phenyl ring, the other three aromatic protons appeared as multiplet at *δ* 8.26–8.28 ppm (methine-H’s and the remaining two Ar–H’s of dimethylaniline ring), plus two singlet signals integrated for one hydrogen each at *δ* 11.22 (NH), 11.35 (NH). Mass spectrum of **5c** displayed a molecular ion peak m/e at 335.94 of its M^+^.

### Potentiodynamic polarization measurements

**Figure 2:**
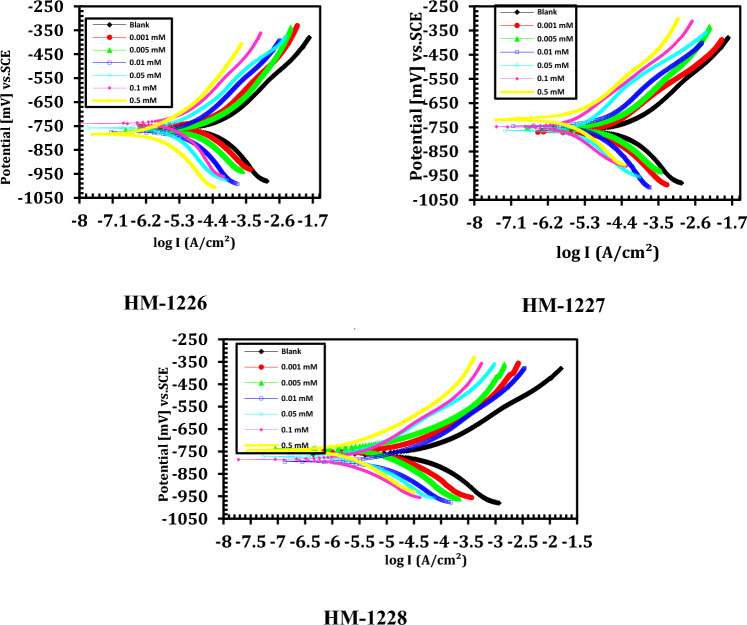
Tafel polarization curves of carbon steel in uninhibited and inhibited oilfield produced water with various concentrations of investigated inhibitors at 298 K.

The polarization curves for API 5L X52 carbon steel in oilfield produced water without and with various doses of inhibitors are shown in Fig. 2 at 25 °C. Electrochemical parameters obtained from PDP curves namely, corrosion current density (i_corr_), corrosion potential (*E*_*corr*_), cathodic and anodic Tafel slopes (βa and βc), corrosion rate (CR) and inhibition effectiveness (${\eta }_{PDP}\%$) were estimated and recorded in Table 3. It is obvious from Table 3 that the corrosion current density considerably decreased in the existence of these investigated inhibitors compared to the blank solution, indicating that all three corrosion inhibitors retarded the corrosion of API 5L X52 carbon steel in oilfield produced water^39,40^.

**Table 3:** Potentiodynamic polarization (PDP) parameters for carbon steel in oilfield produced water with and without inhibitors.

Inhibitor	Conc., (mM)	* E*_corr_ mV (vs. SCE)	*I*_corr._, mAcm^−2^	*β*_a_, mVdec^−1^	*β*_c_, mVdec^−1^	*CR* µm/Y	*IE* %
Blank	–	− 762.5	30.521	193.6	− 148.9	356.9	–
HM − 1228	0.001	− 751.9	15.417	172.1	− 103.7	180.3	49.5
	0.005	− 736.1	11.645	183.5	− 117.3	136.2	61.9
	0.01	− 795.2	9.0675	104.9	− 134.0	106.03	70.3
	0.05	− 773.8	6.239	153.7	− 181.6	72.95	79.6
	0.1	− 786.4	3.625	105.2	− 141.3	42.39	88.1
	0.5	− 745.3	1.592	171.9	− 119.7	18.6	94.8
HM − 1227	0.001	− 770.8	16.527	97.3	− 163.5	193.3	45.9
	0.005	− 753.2	13.376	127.5	− 190.2	156.4	56.2
	0.01	− 746.3	10.764	161.4	− 132.8	125.9	64.7
	0.05	− 764.1	7.0136	170.1	− 159.6	82.0	77.02
	0.1	− 747.8	4.691	204.6	− 147.3	54.9	84.6
	0.5	− 719.6	2.315	136.2	− 125.4	27.1	92.4
HM − 1226	0.001	− 751.4	17.158	145.6	− 181.2	200.6	43.8
	0.005	− 767.3	14.327	164.9	− 135.8	167.5	53.1
	0.01	− 774.6	11.541	177.3	− 146.1	135.0	62.2
	0.05	− 758.2	8.1062	180.4	− 159.5	94.8	73.4
	0.1	− 739.1	5.6417	196.8	− 163.4	66.0	81.5
	0.5	− 784.5	3.152	143.7	− 192.0	36.9	89.7

The obtained findings in Table 3 show that the existence of inhibitors in deep oil wells formation water solution led to small changes of corrosion potential (E_corr_) less than ± 85 mV compared to the inhibitor-free solution. These findings suggest that these inhibitors behave as mixed control-type inhibitors^41,42^. Moreover, the anodic Tafel slopes and the cathodic Tafel slopes were approximately constant and small altered by the dose of inhibitors, which indicated that the prepared inhibitors do effect on the corrosion rate in oilfield produced water and the corrosion mechanism does not change in formation water solution after adding the studied inhibitors. Also, the inhibitor controls both the dissolution of the anodic steel and the cathodic hydrogen evolution reactions by blocking the active reaction sites on metal surface. This could be ascribed to the interaction mechanism between the lone pairs of sulfur, oxygen and nitrogen atoms present in the inhibitors and the empty d-orbital of metal surface via coordination bonds^43–45^.

The inhibition effectiveness (IE%) and surface coverage degree (*ϴ*) from this method was computed by the following expression^46^:

12 $$\eta_{PDP} = \theta \times 100 = \left[ {\frac{{i_{corr}^{0} - i_{corr} }}{{i_{corr}^{0} }}} \right] \times 100$$

where ${i}_{corr}^{0}$ and ${i}_{corr}$ denote the corrosion current density for API X52 carbon steel in oil field produced water without and with inhibitors, respectively. The data in Table 3 exhibited that the inhibition effectiveness obtained using the I_corr_ values rise with the increasing of the inhibitor concentrations and the order of inhibition effectiveness of the inhibitors in oilfield produced water solutions follows the order of: HM-1228˃ HM-1227˃ HM-1226.

### EIS studies

**Figure 3:**
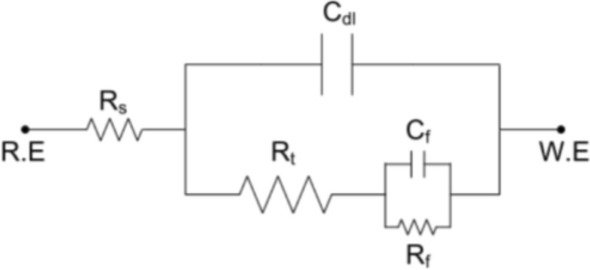
Electrical equivalent circuit utilized to model the impedance data of carbon steel in oil well formation.

**Figure 4:**
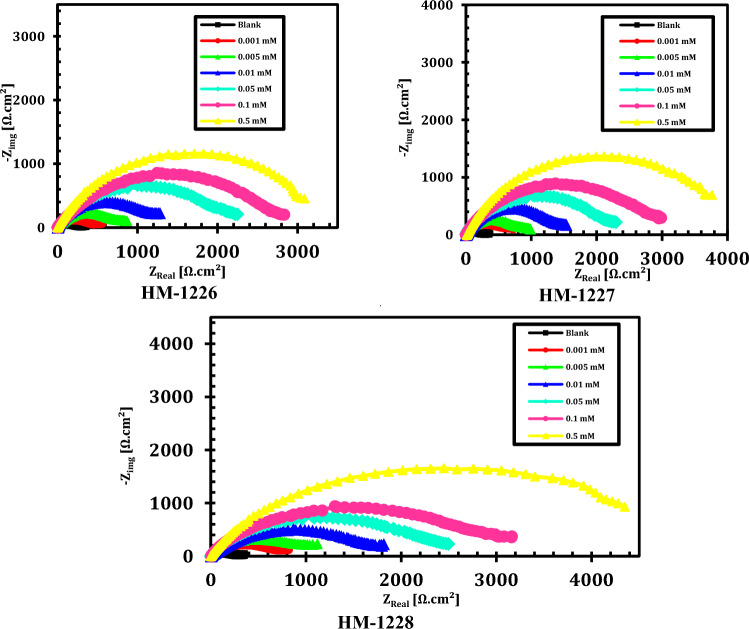
Nyquist plots of carbon steel in oilfield produced water solution without and with various doses of investigated inhibitors at 298 K.

**Figure 5:**
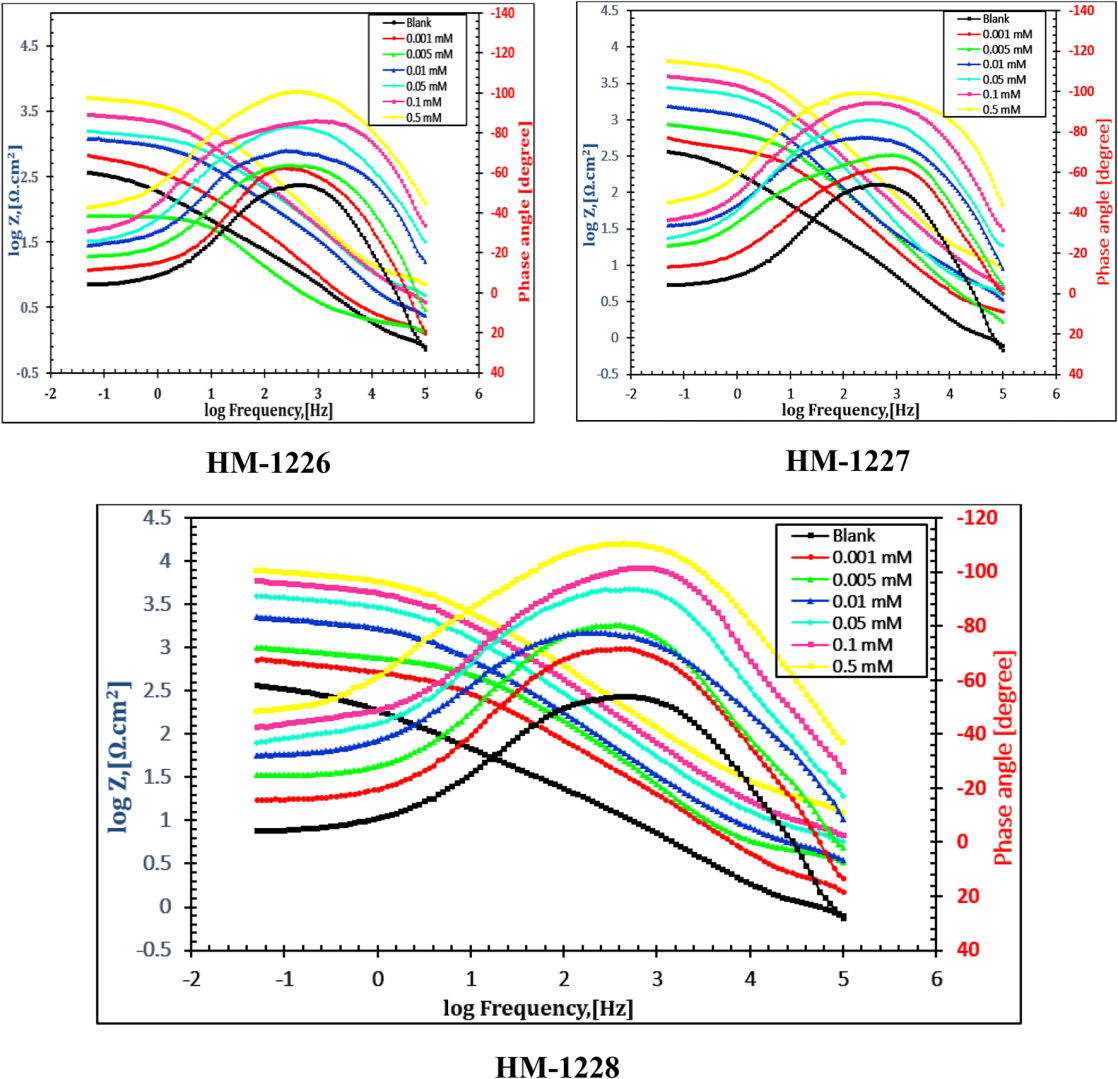
Bode plots of carbon steel in oilfield produced water solution without and with various doses of investigated compounds at 298 K.

The corrosion inhibition of carbon steel in oilfield produced water without and with several doses of inhibitors was performed using EIS method. The electrical equivalent circuit as displayed in Fig. 3 is precisely proposed to fit the EIS measurements (by Z-View Software) consisting of the solution resistance (Rs), the charge transfer resistance(*R*_*ct*_), double-layer capacity (*C*_*dl*_), film resistance (R_f_) and film capacitance (C_f_). Nyquist and bode plots for inhibitors were represented in Figs. 4 and 5, respectively. EIS curves exposed that the impedance response of metal in oilfield produced water was considerably altered after adding the inhibitor molecules. It can be shown that the inhibition efficiency (IE%) and charge transfer resistance (R_c__t_) increase with increasing concentration of the inhibitor. The concentration of the inhibitors does not differ in the form of the EIS figures, suggesting that these inhibitors control the corrosion intereaction activity rather than alter the corrosion inhibition mechanism^47,48^. Moreover, the diameter of impedance improves with the doses of compounds, indicating the adsorption barrier layers of these inhibitors formed on metal in oil field produced water, preventing the dissolution of iron in oilfield produced water^4,22^.

The impedance parameters were recorded in Table 4. The data in Table 4 revealed that R_ct_ values were enhanced with increasing the concentration of the investigated inhibitors, showing improvement in the inhibition performance of these compounds. The value of *C*_*dl*_ was determined from frequency *f*_*max*_ , where the imaginary component of the impedance is maximal (− *Z*_*max*_) by the next Eq. ^49^:

13 $$C_{dl} = \left( {2\pi f_{\max } R_{t} } \right)^{ - 1}$$

**Table 4:** Impedance parameters for carbon steel in oilfield produced water with and without inhibitors.

Inhibitor	Conc., (mM)	*R*s, (Ω cm^2^)	C_f,_ (µF/cm^2^)	*n*_1_	*R*_f_, (Ω cm^2^)	*C*_dl,_ (µF/cm^2^)	*n*_2_	*R*_ct,_ (Ω cm^2^)	*IE* %
Blank	–	6.038	18.92	0.935	11.7	365.9	0.947	311.3	–
HM-1228	0.001	3.524	12.71	0.918	18.9	182.4	0.924	628.1	50.4
	0.005	2.391	9.82	0.893	29.3	159.3	0.914	825.6	62.3
	0.01	5.842	8.34	0.927	42.7	108.2	0.893	1203.7	74.2
	0.05	3.149	7.18	0.904	49.1	71.6	0.965	1881.4	83.5
	0.1	7.159	5.96	0.872	58.2	40.1	0.926	2578.5	87.9
	0.5	6.314	4.23	0.914	67.8	19.8	0.893	5016.2	93.8
HM-1227	0.001	4.517	13.17	0.853	16.4	193.7	0.902	559.2	44.3
	0.005	2.937	10.91	0.924	27.2	168.1	0.819	726.4	57.1
	0.01	2.493	8.63	0.932	39.8	121.4	0.876	1013.8	69.3
	0.05	4.186	7.82	0.862	47.3	79.2	0.914	1541.7	79.8
	0.1	2.078	6.35	0.917	54.6	52.9	0.889	2349.1	86.7
	0.5	3.615	4.72	0.784	63.7	23.5	0.836	3657.9	91.5
HM-1226	0.001	5.402	14.31	0.963	16.1	204.8	0.846	534.1	41.7
	0.005	3.485	11.84	0.885	23.5	176.2	0.913	653.7	52.4
	0.01	5.435	9.45	0.913	35.6	135.7	0.965	870.4	64.1
	0.05	2.597	8.16	0.864	44.2	86.3	0.929	1314.2	76.3
	0.1	4.315	6.52	0.915	51.9	57.4	0.943	1781.9	82.5
	0.5	3.639	5.63	0.846	58.4	32.6	0.817	3314.8	90.6

The inhibition efficency (ƞ%) and surface coaverge ($\theta$) of the inhibitors were calculated by the following expression^50^:

14 $${\varvec{\eta}}_{{{\varvec{EIS}}}} \user2{\% } = \user2{\theta } \times 100 = \left( {1 - \frac{{{\varvec{R}}_{{{\varvec{ct}}}} }}{{{\varvec{R}}_{{{\varvec{ct}}\left( {{\varvec{inh}}} \right)}} }}} \right) \times 100$$

where *R*_*ct*_ and *R*_*ct(inh)*_ are the values of the charge transfer resistance in the absence and presence of these inhibitors, respectively. It is evident from Table 4 that the inhibitor dose values increase the inhibition efficency and the *R*_*ct*_ value, and decrease *C*_*dl*_ value, indicating that the thickness of the ptoective layer developed at the metal/solution interface increases. The tested compounds act as adsorption inhibitors because the *R*_ct_ values for the sample exposed to inhibitors are consistently greater than the *R*_ct_ values without the inhibitor and the *C*_*dl*_ values with the inhibitor are consistently lower than their respective values without the inhibitor. The defensive layer formed on carbon steel surface provides a hindrance in corrosive medium and this barrier increases the inactive surface area that declines the oxidation of the steel surface^51^*.* As a result, *C*_*dl*_ value was reduced upon increasing the dose of inhibitors. This can be described by replacing the water molecules with adsorption of the studied inhibitor compounds that form a protective layer on the metal surface electrode and blocks corrosion reaction sites on metal surface^52–54^.

The data showed that the R_f_ values improve with the increase in the doses of the inhibitors, while the (C_f_) values decrease. These results confirm the establishment of a protective layer of organic inhibitors on metal surface.

The higher inhibition efficacy could be due to the existence of S, O, N atoms, and an aromatic moieties and the use of a compound that provides strong adsorption centres and increases the layer thickness^10^. The order of inhibition effectiveness of the inhibitors in oilfield produced water solutions follows the order of: HM-1228˃ HM-1227˃ HM-1226, which was consistent with the obtained PDP data.

### Adsorption consideration

Adsorption isotherms was followed for understanding the adsorption mode of the tested organic inhibitors on metallic surfaces^55^. The adsorption isotherms can give good insights on the anti-corrosion performance between the carbon steel surface and the organic compounds^56^.

**Figure 6:**
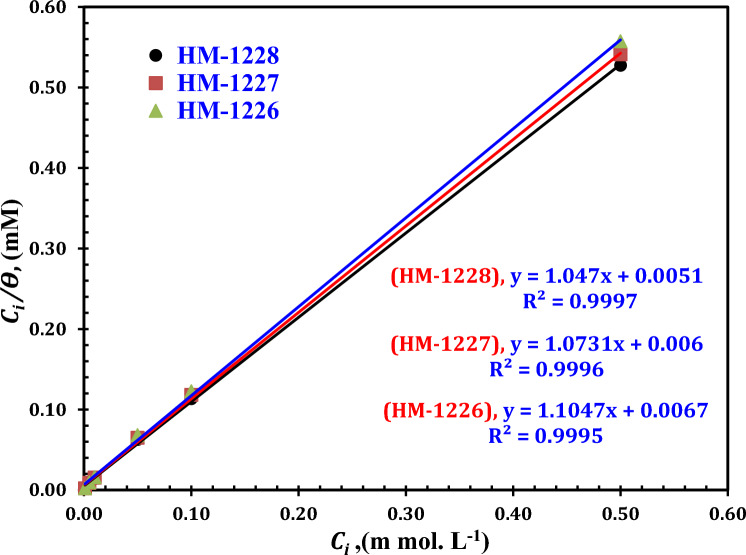
Langmuir adsorption plots for compounds on API 5L X52-type carbon steel in oilfield produced water solution.

The type of adsorption was discriped by plotting, *C*_*i*_*/θ* versus *C*_*i*_ for inhibitors, these curves are represented as shown in Fig. 6. The empirical data revealed that the adsorption behaviour of these inhibitors could best be described by Langmuir adsorption isotherm following the below equation:

15 $$C_{i} /\theta = 1/K_{ads.} + C_{i}$$

where *ϴ* is the degree of surface coverage obtained from polarization data, *K*_*ads*_ is the standard adsorption equilibrium constant and *C*_*i*_ is the molar inhibitor concentration.

The correlation coefficients of these curves and slope values are close to unity, indicating that the inhibition capacity of these investigated organic inhibitors is owing to the ability of these compounds to adsorp on carbon steel surface and the adsorption of these compounds obey the Langmuir adsorption isotherm^57,58^.

The values of K_ads_ were determined from intercepts of the straight lines on the Ci/θ axis and also related to the standard free energy of adsorption $(\Delta G_{ads}^{^\circ } )$ as shown in the following Eq. ^18,59^:

16 $$\Delta G_{ads.}^{^\circ } = - RTln\left( {55.5 K_{ads} } \right)$$

where R is the universal gas constant, T is the absolute temperature (K), and the value 55.5 is the molar concentration of water in solution. The $\Delta G_{ads}^{^\circ }$ and *K*_*ads*_ values of organic inhibitors are recorded in Table 5. The data of K_ads_ data reveal that the strong adsorption of these inhibitors on metal surface in oilfield produced water and the -ve of $\Delta G_{ads}^{^\circ }$ revealed that the adosption process of inhibitors on carbon steel surface is spontaneous^60^. The ∆*G*^◦^_ads_ values revealed that the adsorption of these organic inhibitors on the surface of carbon steel in oilfield produced water is a combination of physical and chemical adsorption^61^.

**Table 5:** Langmuir adsorption parameters for carbon steel in oilfield produced water at 25 °C.

Inhibitor	Regression coefficient (R^2^)	Slope	Intercept	*K*_ads_ (L mol^−1^)	$- \Delta G_{ads}^{^\circ }$ (kJ mol^-1^)
HM-1226	0.9995	1.1047	0.0067	149253.73	39.5
HM-1227	0.9996	1.0731	0.006	166666.67	39.7
HM-1228	0.9997	1.047	0.0051	196078.43	40.1

### Quantum chemical calculations

**Figure 7:**
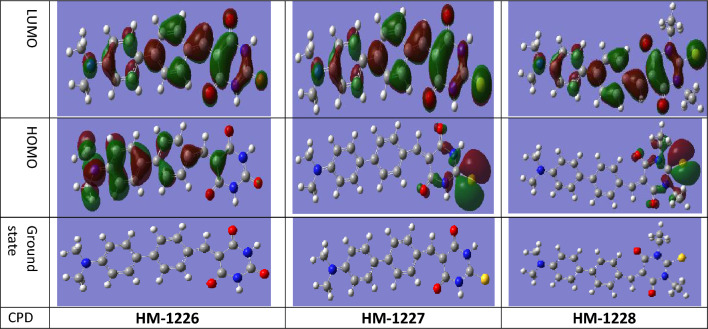
Electron density maps for the HOMO and LUMO of investigated inhibitors.

DFT calculations were computed to explore the chemical and physical properties for the investigated biphenylidene-thiopyrimidine derivatives. The structures of these compounds were first geometry-optimized at ground state in aqueous phase at DFT level. The optimized structures of the studied inhibitors are given in Fig. 7. The frontier molecular orbital (FMO) theory states that the (E_HOMO_) and (E_LUMO_) are important chemical reactivity indices for investigating the reactivity of chemical species in some chemical reactions. The majority of chemical interactions especially processes where adsorption of compounds occur such as lubrication and corrosion inhibitive properties, could be explained by exploring the donor–acceptor interaction between the adsorbed molecules and frontier Molecular orbitals (FMOs) of adsorbent atoms^62^. The increase in the values of E_HOMO_ is often associated with higher capacity of a molecule to donate electrons to an appropriate acceptor molecule which have vacant molecular orbitals^63–65^. In contrast, the lower the E_LUMO_, the more likely that the reacting compound possesses higher capacity to accept the electrons. Thus, when the value of E_LUMO_ is lower, the molecule has higher tendency to gain electrons in particular chemical interactions. The E_HOMO_ can be considered a measure of the ionization potential and the tendency of a species to undergo electrophilic attack. On the contrary, the energy of the LUMO is a measure of the tendency of the molecules to undergo nucleophilic attack. Hence, an enhancement in the tribological, and corrosion inhibition properties of the additives is anticipated with an increasing trend in E_HOMO_, as with the decreasing trend in E_LUMO_. This enhancement in the adsorption of inhibitors on metallic surface is associated with the formation of chemisorbed film. Furthermore, the gap between E_LUMO_ and E_HOMO_ i.e., (ΔE) is an important stability index that was shown to have a correlation with corrosion inhibition potentials in corrosive and tribological systems^62^. The larger ΔE value indicates high molecular stability in a chemical reaction. Furthermore, the ΔE has also been associated with the hardness and softness. When the energy gap (ΔE) is minimum between HOMO and LUMO orbitals of the interacting molecules, it is evident of the soft nature of compounds as ease of polarization is expected. On the other hand, when the energy gap is large, this gives rise to the hard nature of chemical compound. Therefore, the increase in E_HOMO_ and decrease in E_LUMO_ and ΔE is accompanied by an increase in that corrosion inhibition efficiency of compounds^66^. The output of quantum chemical calculations including, E_HOMO_, E_LUMO_ and orbital energy gap (ΔE), dipole moment (μ) is listed in Table 6. Exploring the values of E_HOMO_, E_LUMO_ and ΔE for the studied compounds, the order of inhibition can be anticipated as follows: HM-1228 > HM-1227 >HM-1226. We found that the corrosion inhibition actions of the three studied inhibitors is increasingly consistent with the decreasing order in gap energy, and E_LUMO_, and the higher values µ and ω which characterizes higher reactivity for electrophiles and the higher energy stabilization that occurs when the system obtains an extra electronic charge ∆N from its surroundings. Unlike the trend of some quantum parameters were found insignificantly correlated with the experimental %I.Es. The presence of di-*N*-ethyl groups in HM-1228 increases its electron donating ability as well as enhance lipophilic properties compared to HM-1227 (contain two N–H hydrophilic groups). It is evident that the existence of sulfur atom increases the compound capacity to donate electron through lone pair sharing. On the other side, the donating ability is less in HM-1226 than in HM-1227, due to the lower effect of oxygen atom in donating electrons.

**Table 6:** List of Quantum chemical parameters for the optimized structure of the investigated compounds at DFT.

Code	HM-1226	HM-1227	HM-1228
Program	Gaussian	Gaussian	Gaussian
Method	DFT	DFT	DFT
Basis set	6.31 (d,p)	6.31 (d,p)	6.31 (d,p)
Function	B3LYP	B3LYP	B3LYP
E_HOMO(ev)_	− 8.1959	− 7.4843	− 7.4244
E_LUMO(ev)_	− 6.5676	− 6.5401	− 6.5287
ΔE = E_LUMO_ − E_HOMO_	1.6283	0.9442	0.8958
ƞ = ΔE/2	0.8141	0.4721	0.4479
σ(S) = 1/ƞ	1.2283	2.1182	2.2327
Pi = (E_HOMO_ + E_LUMO_)/2	− 7.3817	− 7.0122	− 6.9766
X = − Pi	7.3817	7.0122	6.9766
Dipole moment (Debye)	11.9734	15.4940	14.1479
∆N (FET)	− 1.5732	− 2.3217	− 2.4074
ω (electrophilicity)	33.4644	52.0762	54.3353
ε (nucleophilicity)	0.0299	0.0192	0.0184
ΔE_Back-donation_	− 0.2035	− 0.1180	− 0.1120

### Molecular simulations

**Figure 8:**
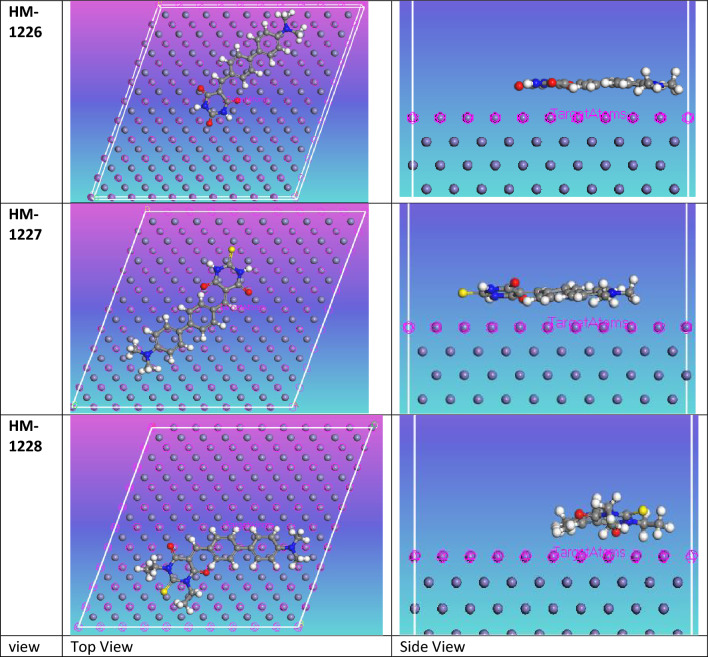
Top and side views for the most stable configuration of inhibitors on metal surface in vacuum conditions.

**Figure 9:**
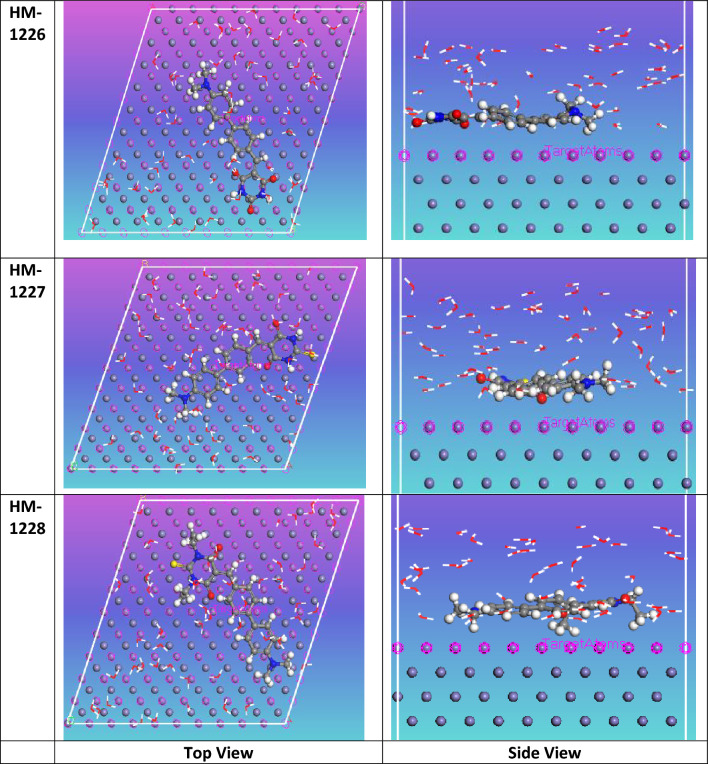
Top and side views for the most stable configuration of biphenylidene-thiopyrimidine inhibitors on metal surface in simulated corrosive media.

In the present study, the interaction of biphenylidene-thiopyrimidine derivatives in aqueous media is explored by Monte Carlo simulation. This method is considered as a powerful tool to provide insights on the equilibrium position of inhibitors on the Fe surface. The most stable optimized structure of inhibitors was introduced in the simulation studies. In order to model part of the corrosive acid environment, the effect of water and corrosive species were also considered in Monte Carlo simulation. Figure 8 displays a top and side view of investigated inhibitor species adsorped on the top surface of the attacked metal in vacuum conditions during simulation process. While the output equilibrium optimized structure of inhibitor compounds on Fe (110) in aqueous media is presented in Fig. 9. It is obvious from both Figs. 8 and 9 that the position of inhibitors is close and in parallel to the metal surface. This gives rise to the adsorption of the inhibitors on corroding metal, implying a potency to form a protective layer, hence protect the metal surface by shielding the metal from the attack of aggressive ions. In tables 7 and 8, the calculated molecular simulation parameters are listed. It can be seen that the adsorption energies of the tested inhibitors are all negative values, suggesting strong interaction occurs between investigated inhibitor compounds with metal surface. The increasing order of adsorption energy of compounds in vacuum conditions was found in agreement with experimental methods HM-1228 > HM-1227 > HM-1226. MC simulations provide useful insights into the performance trend of these inhibitors on the Fe (110) surface. The Inhibitor HM-1228 was the highest in terms of corrosion inhibition due to the presence of two N-substituted –C_2_H_5_ groups on the thiopyrimidine moiety; the existence of electron donating groups is significant in increasing the corrosion inhibition of compounds.

**Table 7:** List of molecular simulation parameters by Monte Carlo method for the biphenylidene- thiopyrimidine derivatives inhibitors in vacuum conditions.

Structures	HM-1226	HM-1227	HM-1228
Total energy	− 342.8224407	− 248.7519043	− 242.8038193
Adsorption energy dEad/dNi	− 194.83319	− 197.9073198	− 226.8900199
Rigid adsorption energy	− 204.3833917	− 207.9916411	− 246.8103204
Deformation energy	9.55020177	10.08432127	19.92030054

**Table 8:** List of molecular simulation parameters by Monte-Carlo method for the inhibitors in simulated corrosive media.

Structures	HM-1226 in water	HM-1227 in water	HM-1228 in water
Total energy	− 3103.698	− 2939.592	− 2993.539
Adsorption energy	− 3011.25	− 2944.288	− 3033.166
Rigid adsorption energy	− 3077.304	− 2993.536	− 3066.8
Deformation energy	66.05402079	49.24766173	33.63460313
Ads energy (Inh): dEad/dNi	− 435.0391568	− 359.1120402	− 361.599735
H_2_O: dEad/dNi	− 71.40595285	− 61.58243207	− 59.9985993

### Surface analysis (AFM)

**Figure 10:**
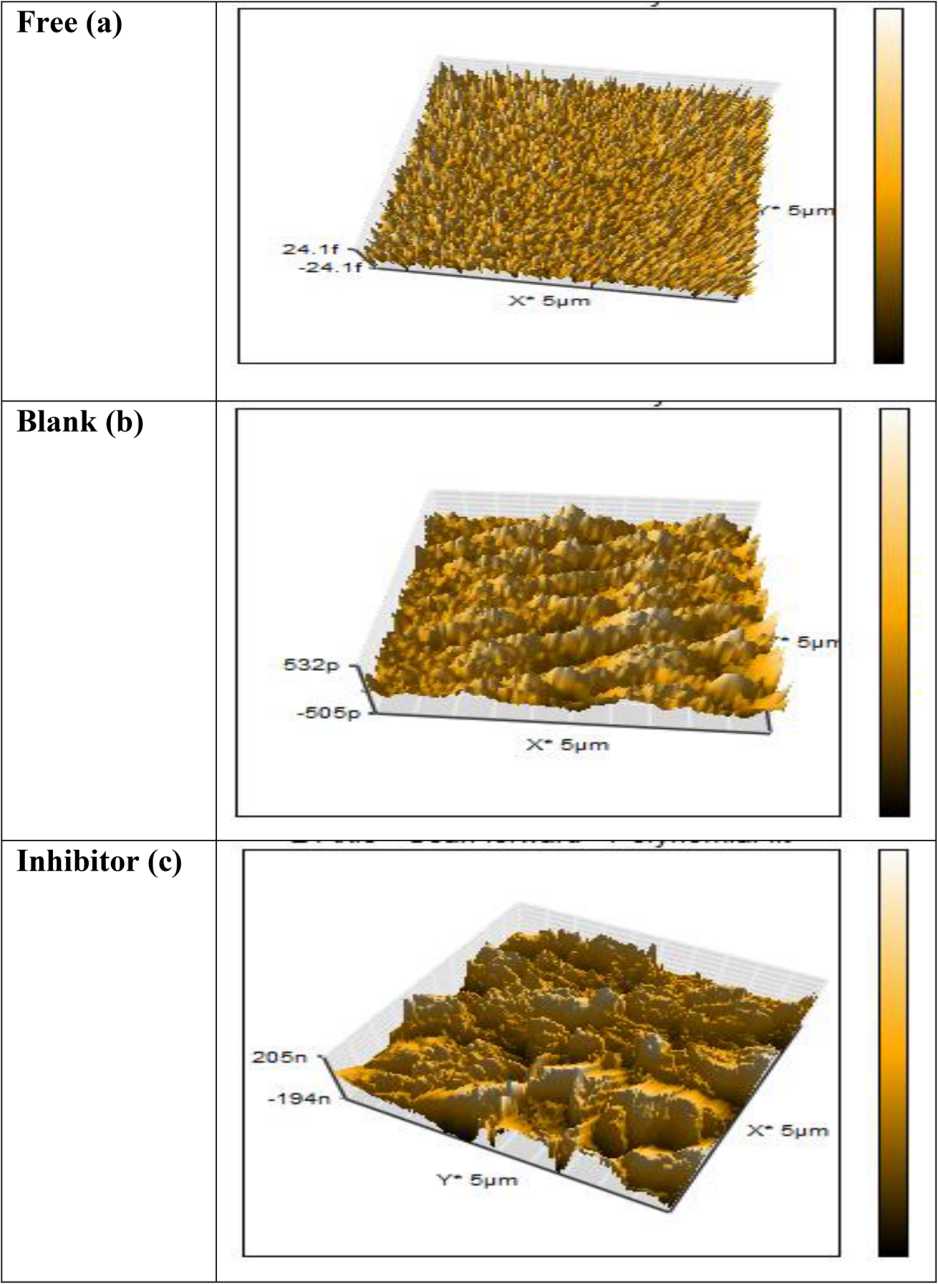
3D AFM images for carbon steel surface in oilfield produced water: (**a**) before immersion, (**b**) in oilfield produced water (blank), and (**c**) in oilfield produced water containing 0.5 mM of HM-1228 inhibitor.

AFM was used to investigate the surface morphology of carbon steel in contact with oilfield-produced water. AFM 3D images of API 5L X52 carbon steel specimens before and after 40 days of immersion in oilfield-produced water without and with 0.5 mM inhibitor (HM-1228) at 25 °C are depicted in Fig. 10a,b,c. AFM image Fig. 10a showed that the morphology of API 5L X52 carbon steel was a very smooth and homogeneous surface^10^. However, API 5L X52 carbon steel form (topography) had severe damage and a rough surface after immersion in oilfield-produced water for 40 days without inhibitor HM-1228 as shown in Fig. 10b. In contrast, the shape of API 5L X52 carbon steel was improved by introducing HM-1228 inhibitor as shown in Fig. 10c and the corrosion degree of carbon steel sample was greatly mitigated. This can be due to the formation of an inhibitive layer/coherent film on API 5L 52X from HM-1228 inhibitor molecules^7,22^. The AFM image revealed that the presence of tested inhibitor (HM-1228) at concentration 0.5 mM in the corrosive solution has an improvement effect on of carbon steel surface, whereas the surface was experienced severe damage when exposed to the corrosive medium without the inhibitor. This explains the maximum inhibition effectiveness of the HM-1228 inhibitor at this concentration.

Furthermore, the average surface roughness (*Ra*) obtained for the polished carbon steel surface was 84.35 nm, while the average surface roughness (*Ra*) in oilfield produced water solution decreased from 571.62 nm (uninhibited surface) to 138.28 nm by introducing 0.5 mM of HM-1228 inhibitor (Inhibited surface), respectively. These results indicated the inhibitory nature of the film formed on the surface of API 5L X52 carbon steel by HM-1228 inhibitor compounds^10,67^.

### Mechanism of corrosion inhibition

**Figure 11:**
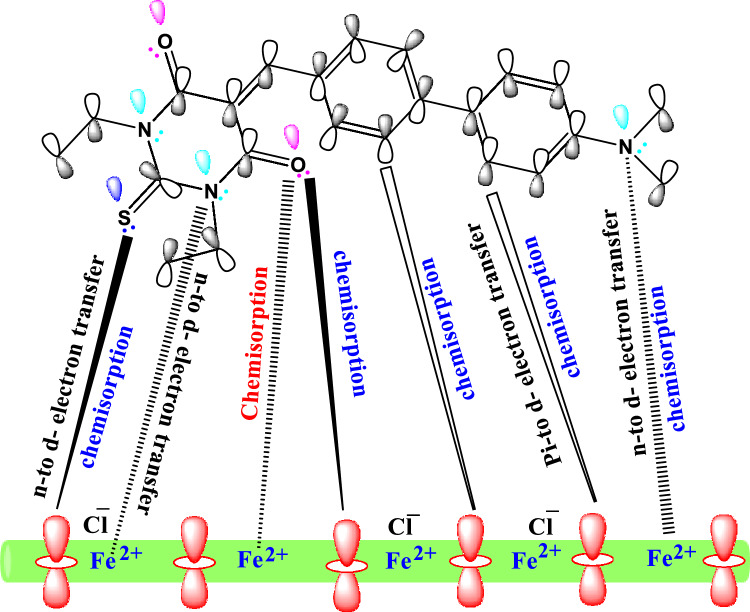
Possible adsorption mechanism of biphenylidene-thiopyrimidine derivative HM-1228 on the carbon steel surface in oil wells formation water.

The biphenylidene-thiopyrimidine derivatives exhibited outstanding corrosion inhibition due to the adsorption of the molecules of these derivatives on the surface of carbon steel using mutual physical and chemical adsorptions in oil wells formation water as explained in Fig. 11. Thiopyrimidine moiety, biphenyl rings, and dimethylamino group are the active centers for adsorption mechanism in the prepared inhibitors. Biphenylidene-thiopyrimidine derivatives can adsorb physically and inhibit the passage of other corrosive ions towards the internal carbon steel surface which impede the corrosion activity. Moreover, these derivatives can be adsorbed chemically on the carbon steel surface through their high electron density cloud, due to structural factors emerging from their possession of S, O, N heteroatoms, π-electrons of aromatic rings, that helped to extend the double bond conjugation throughout the whole structure causing better electron distribution and a more planar conformation on the substrate surface^6^. Therefore, the chemical adsorption was possible via the coordinating bonds between the lone electron pairs located on heteroatoms (N, O, S) of inhibitor molecules and the empty *d*-orbitals of Fe on API 5L X70 type carbon steel surface^18,22^. The previous discussion was consistent with the experimental values of ${\Delta G}_{ads}^{0}$. Accordingly, these derivatives can form a protective adsorbed layer onto the surface of carbon steel via physical and chemical reactions, isolating the carbon steel from further dissolution and corrosion process.

## Conclusions

In conclusion, the research paper presents the synthesis, characterization, and investigation of three novel biphenylidene-thiopyrimidine derivatives as corrosion inhibitors for carbon steel in oilfield produced water. The results demonstrate that these inhibitors exhibit excellent inhibition efficiency, with HM-1228 being the most effective followed by HM-1227 and HM-1226. The polarization curves indicate a decrease in corrosion current density with increasing inhibitor doses, suggesting that these compounds act as mixed type inhibitors. EIS data further support this finding, showing a decline in C_dl_ values and an increase in both Rct and IE% compared to the blank solution. The adsorption of these inhibitors on the carbon steel surface follows Langmuir adsorption isotherm. Additionally, DFT calculation and MC simulations provide insights into the adsorption sites in the inhibitor's molecules, which align well with experimental observations. AFM surface analysis reveals that the inhibitor molecules form a protective layer on the carbon steel surface, effectively insulating it from destructive media. Overall, this research highlights the potential of these biphenylidene-thiopyrimidine derivatives as effective corrosion inhibitors for carbon steel in oilfield produced water Supplemantary Figures S1–S3.

### Supplementary Information

Supplementary Information.

## Data Availability

The published article and its supplementary information files contain all the data generated or analyzed in this study.
