# Supplementary material for: Novel biphenylidene-thiopyrimidine derivatives as corrosion inhibitors for carbon-steel in oilfield produced water
Source: Sci Rep. 2023 Sep 29;13:16388. doi: 10.1038/s41598-023-43312-6 (PMC10541871; doi:10.1038/s41598-023-43312-6)
Supplement: Supplementary file 1 — Supplementary Information. [file 41598_2023_43312_MOESM1_ESM.docx]

**Novel** **Biphenylidene-Thiopyrimidine Derivatives as Corrosion Inhibitors for Carbon-Steel in Oilfield Produced Water**

Hajar A. Ali^a^, Mahmoud M. Shaban^b, *^, Ashraf S. Abousalem^a,c, **^, Eslam A. Ghaith^a^, Abdelaziz S. Fouda^a^, Mohamed A. Ismail^a, ***^

^a^ Chemistry Department, Faculty of Science Mansoura University, Mansoura 35516, Egypt

^b^ Egyptian Petroleum Research Institute, Nasr City 11727, Cairo, Egypt

^c^ Quality Control Laboratory, Operations Department, Jotun, Egypt

**Supplementary Material**

**I. Figures**

**1.1. Figures S1: IR Spectra of the new biphenyl-pyrimidines 5a-c.**

Infrared (IR) spectra were recorded using KBr wafer technique on a Thermo scientific Nicolet iS10 FT-IR Spectrometer.

**1.2. Figures S2: NMR Spectra of the new biphenyl-pyrimidines 5a-c.**

A JEOL spectrometer was used for recording ^1^H-NMR (500 MHz) and ^13^C-NMR (125 MHz) spectra and chemical shifts (*δ*) were measured in parts per million (ppm) relative to the used DMSO-*d_6_* as solvents and self-internal standard.

**1.3. Figures S3: Mass Spectra of the new biphenyl-pyrimidines 5a-c.**

Electron impact mass spectra were determined at 70 eV on Varian MAT 311Kratos instrument.

**1.1. Figures S1: IR Spectra of the new biphenyl-pyrimidines 5a-c**

| ****    **Compound HM-1228: IR Spectrum** |
| --- |

| ****    **Compound HM-1227: IR Spectrum** |
| --- |

| ****    **Compound HM-1226: IR Spectrum** |
| --- |

**1.2. Figures S2: NMR Spectra of the new biphenyl-pyrimidines 5a-c**

|     **Compound HM-1228:** ^1^H-NMR/JEOL 500 MHz (DMSO-*d*_6_) |
| --- |

| 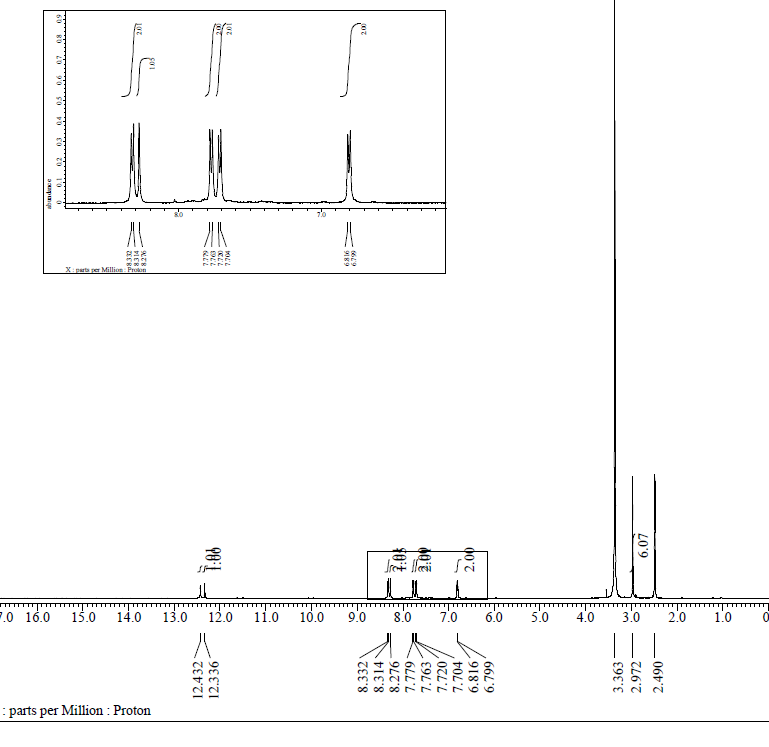    **Compound HM-1227:** ^1^H-NMR/JEOL 500 MHz (DMSO-*d*_6_) |
| --- |

| 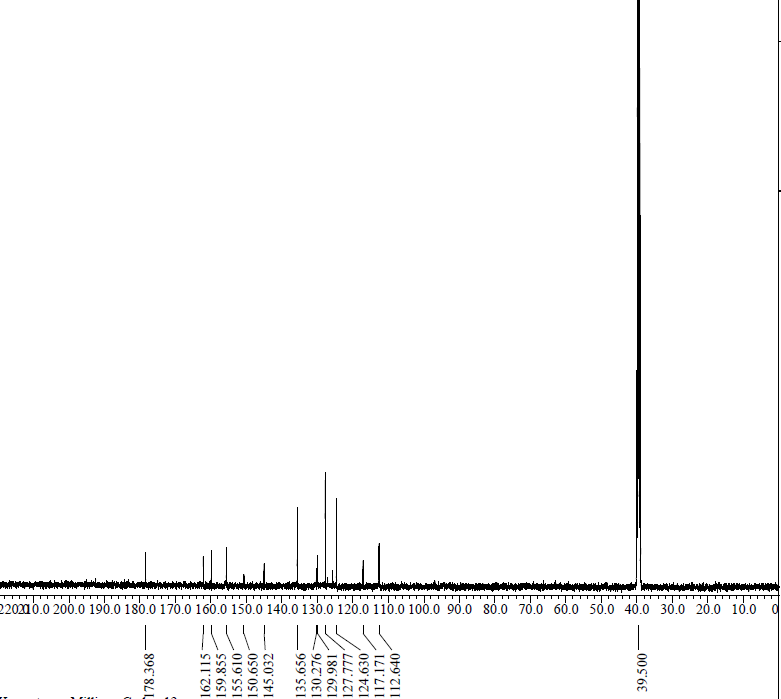    **Compound HM-1227:** ^13^C-NMR/JEOL 125 MHz (DMSO-*d*_6_) |
| --- |

| 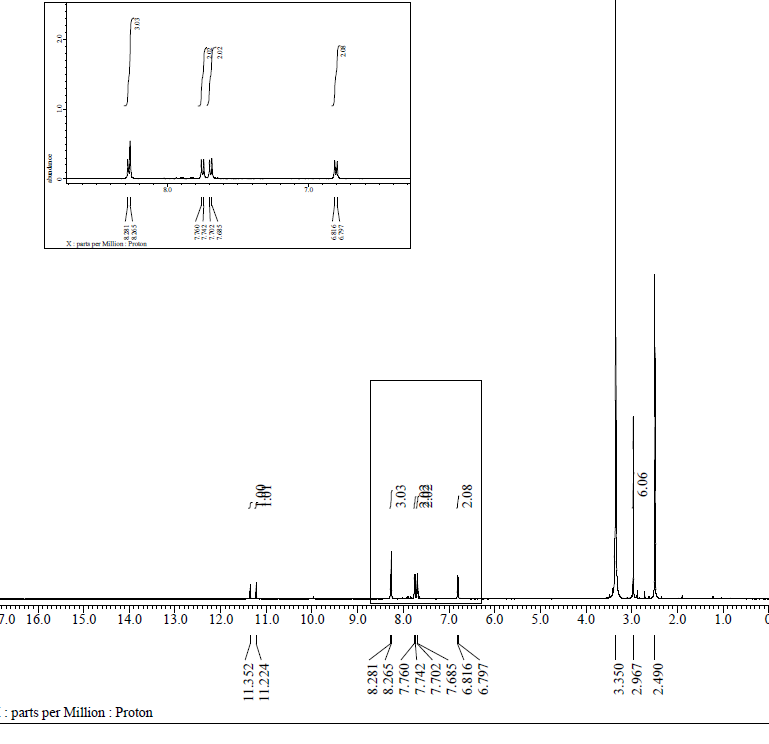    **Compound HM-1226:** ^1^H-NMR/JEOL 500 MHz (DMSO-*d*_6_) |
| --- |

**1.3. Figures S3: Mass Spectra of the new biphenyl-pyrimidines 5a-c**

|     **Compound HM-1228: Mass Spectrum** |
| --- |

|     **Compound HM-1227: Mass Spectrum** |
| --- |

|     **Compound HM-1226: Mass Spectrum** |
| --- |
